# Mental health problems, sleep quality and overuse injuries in advanced Swedish rock-climbers – the CLIMB study

**DOI:** 10.1186/s13102-024-00826-4

**Published:** 2024-02-12

**Authors:** Fredrik Identeg, Isabel Nigicser, Klara Edlund, Niklas Forsberg, Mikael Sansone, Ulrika Tranaeus, Henrik Hedelin

**Affiliations:** 1 Division of Psychology, Department of Clinical Neuroscience, Karolinska Institutet, SE-171 77 Stockholm, Sweden; 2 Musculoskeletal and Sports Injury Epidemiology Center, Department of Health Promotion, Sciences, Sophiahemmet University, Stockholm, Sweden; 3 Department of Orthopedics, Institute of Clinical Sciences, Sahlgrenska Academy, University of Gothenburg, Gothenburg, Sweden; 4 Department of Physiology, Nutrition, Biomechanics, Sport Performance & Exercise Research & Innovation Center - Stockholm, SPERIC-S, The Swedish School of Sport and Health Sciences, Stockholm, Sweden; 5 Unit of Intervention and Implementation for Worker Health, Institute of Environmental Medicine, Karolinska Institutet, Stockholm, Sweden; 6 Independent Researcher, Stockholm, Sweden

**Keywords:** Rock climbing, Mental health, Anxiety, Depression, Sleep disturbances, Overuse injury

## Abstract

**Objectives:**

To examine the prevalence of mental health problems (depression, anxiety, and stress), sleep quality, and disability due to overuse injuries in advanced and elite rock-climbers. The rock-climbers were compared to a group of non-climbing controls.

**Methods:**

A self-selected sample of advanced and elite Swedish rock-climbing athletes was recruited through the Swedish Rock-climbing Federation, local rock-climbing gyms and through social media. A control group, matched in size was recruited. Participants in the control group answered an online survey of validated questionnaires, examining symptoms of stress, anxiety, depression, sleep quality. The climbing participants answered the same survey as the non-climbing controls but with additional questions regarding musculoskeletal problems and disabilities related to these. Outcome measures used were the Depression Anxiety Stress Scale, Pittsburgh Sleep Quality Index and The Oslo Sports Trauma Research Center Overuse Injury Questionnaire.

**Results:**

A total of 183 participants were included in the rock-climbing group, and 180 participants in the control group. In the rock-climbing group the mean age (SD) was 28.2 (8.3) years among women and 30.5 (9.6) years in men. The mean BMI of women was 21.2 (2.2) and 22.8 (2.1) in men. A total of 30.6% of the rock-climbing group (26.7% of men, 35.9% of women) reported at least moderate levels of symptoms of depression and 23.1% (17.2% men, 30.8% women) at least moderate levels of symptoms of anxiety. A total of 48.4% of rock-climbers (39.1% men, and 61.6% women) reported at least moderate levels of symptoms of stress. Among the rock-climbers, 45.0% reported having poor sleep quality. There were no statistical significant differences (*p* = 0.052–0.96) in mental health problems or sleeping problems between the rock-climbers and the controls. Among rock-climbers, reports of one-week prevalence of injury related problems was: Finger and hand (49.5%), Shoulder (35.2%), Knee (29.1%), Lumbar back (26.4%), Arm (25.3%), Thoracic back and neck (17.0%), and Foot and lower leg (12.1%).

**Conclusion:**

The overall results indicate high levels of symptoms of mental health problems and poor sleep quality in both rock-climbers and controls. Although no significant differences between the climbing group and the control group was displayed, symptoms that warrant clinical attention is high. Overuse injuries were commonly reported among the rock-climbers in all examined injury locations. Previous studies reporting mental health problems to be more prevalent among athletes were contradicted in this study. The results display the need for a broader perspective regarding climbers general health and the need to provide structured care and adequate support in order to come to terms with these concerns.

## Introduction

Mental health problems including depression, anxiety, stress, and sleep disturbances are a growing concern in the field of sports medicine [1]. Research has shown a high prevalence of mental health problems among athletes [2–5]. Although physical activity has proven beneficial for the mental wellbeing among the general population [6], elite level exercise and training are suggested to increase the risk for mood disorders [7]. While mental health problems are a common concern of epidemic measures in the general population, the risk factors for the development of these problems among athletes may differ from non-sports environment [1]. Among athletes, the sport specific environment with publicity and pressure to perform are proposed risk factors for developing mental health problems [8]. Similarly, injured athletes suffering from sport related injuries and athletes who experience musculoskeletal pain are more prone to suffer from concurrent mental health problems [8].

Rock-climbing has grown in popularity in recent years, developing into a more commercial and competitive nature, with growing media coverage. Research regarding the potential risk of this development has however been lacking. Little is known about mental health problems in rock-climbers, apart from disordered eating, which has been a topic of discussion in the climbing community throughout the years. A few studies raising concerns about weight sensitivity, lean body ideals, body dissatisfaction have also been published [9, 10]. Mental health problems are of escalating global measures, and specific sport-related factors may play an important role, both as risk- and protective factors for the development of ill health in athletes. Due to the increasing number of people practicing rock-climbing, it is a necessity to bridge the knowledge gap regarding mental health, overuse injuries and a safe sport environment to ensure a sustainable growth of the sport. Regarding musculoskeletal injury and pain, an important variable for mental well-being in athletes, multiple studies have reported on the injury prevalence and incidence in rock-climbing, but few have explored the subjective disability related to injury in rock-climbers [8, 11]. Given the physical and mental demands of advanced rock-climbing, and the well-known risk factors for mental ill health in athletes in general, the prevalence of these health issues among rock-climbers need further scientific mapping.

The aim of this study was to present the prevalence of symptoms of depression, anxiety, stress, sleeping disturbances, as well as functional impairment related to injury among high-level rock-climbers. Furthermore, we aimed to study differences between rock-climbers and non-rock-climbing controls with regards to these variables.

## Methods

### Participants

Swedish rock-climbers were prospectively invited to participate in the study through completing a web based survey. The survey was open for registration between February 14th and April 14th, 2022. With the aim of recruiting a comprehensive sample of participants, information regarding the study was distributed to the Swedish Rock-climbing Federation, to local rock-climbing gyms of Sweden, and to the senior and junior national teams of Sweden. The study was also promoted through relevant, rock-climbing-related social media channels, including Facebook and Instagram.

The inclusion criteria for the rock-climbing group were: minimum of 13 years of age and rock-climbing at an advanced, elite, or higher elite level according to the International Rock-climbing Research Association (IRCRA) [12]. For men, this consisted of having completed a non-flash boulder route of grade 6B+ or harder (Font grade) or a red-point lead route of grade 7a + or above (French grade) within the last year. For women this included having completed a non-flash boulder route of grade 6A or harder or a red-point lead route graded 6c or above within the last year. Only participants living in Sweden were included. Since the study aimed to recruit a representative sample of the whole eligible rock-climbing population, no exclusion criteria were set.

Participants of the control group were recruited through social media, and through advertisement at the University of Gothenburg and Chalmers University of Technology (Gothenburg). Inclusion criteria for the control group, consisted of a minimum of 13 years of age. Exclusion criteria consisted of prior or current participation at an elite level in any sport and current or previous extensive experience of rock climbing.

No previous data on self reported disability related to overuse injuries exists for rock-climbers. Based on previous retrospective data from rock-climbers, using a power of 80%, a significance level of 5%, and an expected relative risk of 1.5 for the primary outcome, we calculated that at least 55 participants were needed in each group (rock-climbers and control group) [13]. The study constitutes a baseline measurement, as part of a larger longitudinal study, with pre-registered procotol in BMJ OPEN, where a more detailed description of the study methodology can be found [14].

### Questionnaires

Data was collected through a web-based survey with well-used and validated questionnaires. All used questionaires are referenced below, and were answered separately as intended by the original authors. Self-reported demographic information regarding biological sex, age, Body Mass Index (BMI), and training volume was recorded. Self-reported rock-climbing ability was evaluated by reporting the three most difficult non-flash^1^ ascents in the last 3 months, flash ascents of indoor and outdoor boulder routes, as well as sport rock-climbing routes in the last 12 months, according to IRCRA and Draper et al. [12, 15]. Preferred rock-climbing discipline, training volume in each rock-climbing discipline, competition frequency, and total years of rock-climbing was collected according to the recommendations of IRCRA [12].

Symptoms of mental health problems was assessed using the *Depression Anxiety Stress Scale (DASS-21)* [16]. To measure sleep quality, the *Pittsburg sleep quality Index (PSQI)* including 19 items addressing the seven subscales of: Sleep duration, efficiency, quality, latency, medication use, disturbances, and daytime dysfunctions were used [17]. According to Buysse et al., PSQI Global score was dichotomized, where participants who had a score of > 5 were classified as “poor sleepers”, and those with a score of ≤5 were classified as “good sleepers” [18].

Self-reported experience of musculoskeletal problems and overuse injuries was only examined in the rock-climbing group. *The Oslo Sports Trauma Research Center Overuse Injury Questionnaire (OSTRC-O)* was used to measure athlete’s self-reported participation in sport, as well as modified training/competing, pain, and decreased performance in relation to overuse injury [19]. Apart from the standardized injury locations included in the OSTRC-O, the questionnaire was expanded to survey injury locations common in rock-climbing, including the shoulder, arm, hand and finger, back (lumbar, thoracic, and cervical), and lower leg and foot.

To measure disability associated with injury, all injury locations were reported as “any problems” or “substantial problems”. According to Larsen et al. [19], we defined “any problems” as reporting one or above of the following on the 4 OSTRC-O questions: (1) full participation or less, but with problems (2); reduction in training volume to any extent (3); affected performance to any extent; and (4) pain experienced in relation to sport participation. “Substantial problems” were defined as at least one of the following answer options to the OSTRC-O questions: (1) moderate or severe reduction in training volume (2); moderate or severe affected performance; and (3) inability to participate.

### Statistical analysis

The IBM SPSS Statistics for MAC, version 24 (IBM Corp., Armonk, N.Y., USA) was used for demographic description of the data and statistical tests were performed using the statistical analysis software SAS 9.4 for Windows (SAS Institute Inc., Cary, NC, USA). The level of significance was set at *p* <  0.05. Nominal values were presented as n (%), and continuous variables were presented as mean (standard deviation, SD) when normally distributed, and median (Interquartile Range, IQR) when skewed. For comparison between groups, Fisher’s Exact test (lowest 1-sided *p*-value multiplied by 2) was used for dichotomous variables and the Mantel-Haenszel Chi Square Exact test was used for ordered categorical variables.

## Results

### Demographics

A total of 183 participants were included in the rock-climbing group, and 180 participants in the control group. The mean age (SD) of women in the rock-climbing group was 28.2 (8.3) years and 30.5 (9.6) years for men. In the rock-climbing group, the mean BMI of women participants was 21.2 (2.2), and in men 22.8 (2.1) (Table 1). There was a difference in BMI between rock-climbers and controls both among women and men, and further a difference in age was observed for male athletes and controls.

**Table 1:** Demographics of rock-climbers and controls. Data is presented as mean (SD)

	Total			Men			Women		
	Rock-climbers (*n* = 183)	Controls (*n* = 180)	*p*-value	Rock-climbers (*n* = 105)	Controls (*n* = 71)	*p*-value	Rock-climbers (*n* = 78)	Controls (*n* = 109)	*p*-value
**Age**	29.5 (9.1)	29.0 (8.9)	0.57	30.5 (9.6)	27.3 (6.8)	0.012*	28.2 (8.3)	30.0 (9.9)	0.18
**BMI**	22.1 (2.3)	23.3 (4.1)	< 0.001*	22.8 (2.1)	23.8 (3.8)	0.018*	21.2 (2.2)	22.9 (4.3)	< 0.001*

* = significant *p*-value

A total of 135 participants in the rock-climbing group were classified as advanced rock-climbers, according to IRCRA, while 47 were classified as elite or higher elite. The mean age of the advanced rock-climbing group was 30.8 (9.0) and the elite and higher elite group 25.8 (8.4). The mean BMI of participants in the advanced group was 22.4 (2.2), and in the elite and higher elite group 21.2 (2.1). The advanced group had been rock-climbing for a median of 4 (3;9) years and were training at an average of 3 [1] rock-climbing sessions per week, with 2 (2;3) rock-climbing hours per session. The elite and higher elite group had been rock-climbing for a median of 8 (5;12) years and were training at an average of 4 [1] sessions per week, with 3 (2;3) rock-climbing hours per session (Table 2).

**Table 2:** Characteristics of the rock-climbing group. Data is presented as mean (SD) when normally distributed and median (IQR) when skewed. IRCRA = International Rock Climbing Research Association

	Advanced (*n* = 135)			Elite and higher elite (*n* = 47)		
	Total	Men	Woman	Total	Men	Women
**Age**	30.8 (9.0)	32 (9)	29 (8)	25.8 (8.4)	26 (9)	26 (8)
**Height**	175 (9)	180 (6)	167 (7)	171 (11)	179 (9)	164 (8)
**Weight**	69 (10)	74 (7)	61 (8)	63 (13)	72 (11)	54 (7)
**BMI**	22.4 (2.2)	22.9 (2.1)	21.7 (2.2)	21.2 (2.1)	22.2 (2.0)	20.2 (1.8)
**Avarage IRCRA grade**	20 (2)	21 (1)	19 (1)	24 (2)	25 (1)	23 (2)
**Years rock-climbing**	4 (3;9)	5 (2;10)	4 (3;7)	8 (5;12)	8 (6;12)	9 (5;14)
**Rock-climbing sessions per week**	3 (1)	3 (1)	3 (1)	4 (1)	4 (1)	4 (1)
**Rock-climbing hours per session**	2 (2;3)	2 (2;3)	3 (2;3)	3 (2;3)	3 (2;3)	3 (2;4)

### Mental ill health

A total of 30.6% of the rock-climbing group (26.7% of men, 35.9% of women) reported moderate or more severe symptoms of depression (DASS-21), compared to 28.2% (28.2% of men and 28.4% of women) in the control group. A total of 23.1% of the rock-climbing group (17.2% of men, 30.8% of women) reported moderate or more severe symptoms of anxiety (DASS-21), compared to 26.6% (21.2% of men, 30.3% of women) in the control group. A total of 48.4% of the rock-climbing group (39.1% men, 61.6% women) reported moderate or more severe levels of symptoms of stress (DASS-21), compared to 45.3% (39.5% of men and 48.6% of women) of the control group. Regarding sleep quality (PSQI), 54.6% (61.0% men, 46.2% women,) were categorized as good sleepers, compared to 65.0% (70.4% men, 61.5% women) in the control group (Table 3). There were no differences observed in reported symptoms of depression, anxiety, stress or sleep quality between the rock-climbing and control group (*p* = 0.2–0.9). Similarly, there were no differences between elite and advanced rock-climbers, as far as reported symptoms of depression, anxiety, stress or sleep quality (Table 4).

**Table 3:** Differences in mental health problems between rock-climbers and controls, presented as total and separated by sex. Data is presented as n (%)

	Total			Men			Women		
	Rock-climbers (*n* = 183)	Controls (*n* = 180)	*p*-value	Rock-climbers (*n* = 105)	Controls (*n* = 71)	*p*-value	Rock-climbers (*n* = 78)	Controls (*n* = 109)	*p*-value
**DASS Depression Score**			0.69			0.71			0.20
**Normal**	104 (57.1)	110 (60.8)		64 (61.0)	45 (63.4)		40 (51.3)	65 (59.6)	
**Mild**	23 (12.6)	19 (10.5)		13 (12.4)	6 (8.5)		10 (12.8)	13 (11.9)	
**Moderate**	33 (18)	23 (12.7)		19 (18.1)	8 (11.3)		14 (17.9)	15 (13.8)	
**Severe**	4 (2.2)	15 (8.3)		2 (1.9)	7 (9.9)		2 (2.6)	8 (7.3)	
**Extremely Severe**	19 (10.4)	13 (7.2)		7 (6.7)	5 (7.0)		12 (15.4)	8 (7.3)	
**DASS Anxiety Score**			0.25			0.26			0.96
**Normal**	129 (70.9)	121 (66.9)		82 (78.1)	53 (74.6)		48 (61.5)	67 (61.5)	
**Mild**	11 (6.0)	12 (6.6)		5 (4.8)	3 (4.2)		6 (7.7)	9 (8.3)	
**Moderate**	24 (13.2)	24 (13.3)		11 (10.5)	6 (8.5)		13 (16.7)	18 (16.5)	
**Severe**	8 (4.4)	5 (2.8)		4 (3.8)	2 (2.8)		4 (5.1)	3 (2.8)	
**Extremely Severe**	10 (5.5)	19 (10.5)		3 (2.9)	7 (9.9)		7 (9.0)	12 (11.0)	
**DASS Stress score**			0.61			0.95			0.053
**Normal**	65 (35.7)	73 (40.3)		47 (44.8)	38 (53.5)		18 (23.1)	35 (32.1)	
**Mild**	29 (15.9)	26 (14.4)		17 (16.2)	5 (7.0)		12 (15.4)	21 (19.3)	
**Moderate**	46 (25.3)	40 (22.1)		29 (27.6)	18 (25.4)		18 (23.1)	21 (19.3)	
**Severe**	14 (7.7)	15 (8.3)		9 (8.6)	3 (4.2)		5 (6.4)	12 (11.0)	
**Extremely Severe**	28 (15.4)	27 (14.9)		3 (2.9)	7 (9.9)		25 (32.1)	20 (18.3)	
**Pittsburg Sleep Quality Index**			0.26			0.054			0.052
**Good sleepers**	100 (54.6)	117 (65.0)		64 (61.0)	50 (70.4)		36 (46.2)	67 (61.5)	
**Poor sleepers**	83 (45.4)	63 (35.0)		41 (39.0)	21 (29.6)		42 (53.8)	42 (38.5)	

*DASS* Despression Anxiety Stress Scale

**Table 4:** Differences in mental health problems between advanced and elite rock-climbers. Data is presented as n (%)

	Advanced (*n* = 135)	Elite and Higher Elite (*n* = 47)	*p*-value
**DASS Depression Score**			0.26
**Normal**	72 (53.3)	32 (68.1)	
**Mild**	21 (15.6)	2 (4.3)	
**Moderate**	23 (17.0)	9 (19.1)	
**Severe**	4 (3.0)	0	
**Extremely Severe**	15 (11.1)	4 (8.5)	
**DASS Anxiety Score**			0.94
**Normal**	95 (70.4)	34 (72.3)	
**Mild**	9 (6.7)	2 (4.3)	
**Moderate**	18 (13.3)	6 (12.8)	
**Severe**	5 (3.7)	3 (6.4)	
**Extremely Severe**	8 (5.9)	2 (4.3)	
**DASS Stress Score**			0.38
**Normal**	54 (40.0)	11 (23.4)	
**Mild**	19 (14.1)	10 (21.3)	
**Moderate**	32 (23.7)	14 (29.8)	
**Severe**	7 (5.2)	7 (14.9)	
**Extremely Severe**	23 (17.0)	5 (10.6)	
**Pittsburg Sleep Quality Index**			0.61
**Good sleepers**	76 (56)	24 (51)	
**Poor sleepers**	59 (44)	23 (49)	

*DASS* Depression Anxiety Stress Scale

### Musculoskeletal problems among rock-climbers

The injury location with the highest one-week prevalence of any musculoskeletal problems was the hand and fingers (49.5%). Of all rock-climbers, 16.5% experienced substantial finger and hand problems. Apart from finger and hand related problems, the prevalence of musculoskeletal problems of all recorded body location, was in descending order; Shoulder (35.2%), Knee (29.1%), Lumbar back (26.4%), Arm (25.3%), Thoracic back and neck (17.0%) and Foot and lower leg (12.1%). Detailed descriptions for advanced and elite rock-climbers specified by gender, is displayed in Table 5.

**Table 5:** Prevalence of musculoskeletal problems among advanced, and elite and higher elite rock-climbers, presented in total and separated by sex. Data is presented as n (%)

	Total (182)			Advanced (*n* = 135)			Elite and higher elite (*n* = 47)		
	Total	Woman (*n* = 53)	Men (*n* = 82)	Total	Woman (*n* = 53)	Men (*n* = 82)	Total	Women (*n* = 23)	Men (*n* = 24)
Any foot and lower leg problems	22 (12.1)	8 (10.4)	14 (13.3)	17 (12.6)	7 (13.2)	10 (12.2)	5 (10.6)	1 (4.2)	4 (17.4)
Substantial foot and lower lack problems	8 (4.4)	3 (3.9)	5 (4.8)	6 (4.4%)	2 (3.8%)	4 (4.9)	2 (4.3)	1 (4.2)	1 (4.3)
Any knee problems	53 (29.1)	24 (31.2)	29 (27.6)	39 (28.9)	15 (28.3)	24 (29.3)	14 (29.8)	9 (37.5)	5 (21.7)
Substantial knee problems	8 (4.4)	5 (6.5)	3 (2.9)	5 (3.7)	3 (5.7)	2 (2.4)	3 (6.4)	2 (8.3)	1 (4.3)
Any lumbar back problems	48 (26.4)	22 (28.6)	26 (24.8)	30 (22.2)	9 (17.0)	21 (25.6)	18 (38.3)	13 (54.2)	5 (21.7)
Substantial lumbar back problems	9 (4.9)	4 (5.2)	5 (4.8)	5 (3.7)	2 (3.8)	3 (3.7)	4 (8.5)	2 (8.3)	2 (8.7)
Any thoracic back and neck problems	31 (17.0)	18 (23.4)	13 (12.4)	19 (14.1)	11 (20.8)	8 (9.8)	12 (25.5)	7 (29.2)	5 (21.7)
Substantial thoracic back and neck problems	6 (3.3)	2 (2.6)	4 (3.8)	2 (1.5)	0 (0)	2 (2.4)	4 (8.5)	2 (8.3)	2 (8.7)
Any shoulder problems	64 (35.2)	29 (37.7)	35 (33.3)	44 (32.6)	18 (34.0)	26 (31.7)	20 (42.6)	11 (45.8)	9 (39.1)
Substantial shoulder problems	20 (11.0)	9 (11.7)	11 (10.5)	12 (8.9)	4 (7.5)	8 (9.8)	8 (17.0)	5 (20.8)	3 (13.0)
Any arm problems	46 (25.3)	11 (14.3)	35 (33.3)	36 (26.7)	9 (17.0)	27 (32.9)	10 (21.3)	2 (8.3)	8 (34.8)
Substantial arm problems	14 (7.7)	6 (7.8)	8 (7.6)	11 (8.1)	5 (9.4)	6 (7.3)	3 (6.4)	1 (4.2)	2 (8.7)
Any finger and hand problems	90 (49.5)	35 (45.5)	55 (52.4)	64 (47.4)	20 (37.7)	44 (53.7)	26 (55.3)	15 (62.5)	11 (47.8)
Substantial finger and hand problems	30 (16.5)	9 (11.7)	21 (20.0)	21 (15.6)	5 (9.4)	16 (19.5)	9 (19.1)	4 (16.7)	5 (21.7)

## Discussion

The main results of this study display a markedly high prevalence among rock-climbers of clinically relevant (DASS score of moderate or above) symptoms of depression (30.6%), anxiety (23.1%), stress (48.4%) and sleeping disturbances (45.4%) as compared to the normative material for the relevant scores. However, there were no differences in mental ill health between the rock-climbing group and the control group, attributed to the similarly high scores displayed in the control group. It was also noticeably common among rock-climbers at this level to report subjective problems related to injury, with the OSTRC-O 1 week prevalence ranging from 10 to 50% depending on body location.

To our knowledge, only one study has been previously published regarding the prevalence of mood disorders among climbers [20]. The study examined 113 climbers in Poland, and showed a prevalence of 27% for both mild depression and moderate anxiety. The results are thus in line with the present study. Comparing the results to normative materials, not exclusively related to a sports environment, the numbers could be compared with a Spanish sample of college students (*n* = 1074) where the number of students scoring moderate levels of symptoms of depression, anxiety and stress (DASS-21) was considerably lower (depression: 18.4%; anxiety: 23.6% and stress: 34.5%) [21]. Although not directly comparable due to differences in reporting, the Swedish national public health survey of 2022 provided by the Public Health Agency of Sweden, similarly displayed lower levels of mental health problems in people of age 16–29 (Severe Anxiety; 15,7%, Stress; 27%, mild and severe sleep disturbances; 39%). Comparing to other sports, a systematic review and meta-analysis by Gouttebarge et al. in 2019 examining mental ill health among athletes, included 9 studies, and displayed that a mean of 33.6% (95% CI: 27.4–39.7) of elite athletes, reported symptoms of either depression or anxiety [22]. The same study reported that 20.9% (95% CI: 15.2–26.6) of participants reported symptoms of sleeping disturbances [22]. Our results are thus in line, although appear to be of a higher severity, as compared to other sports.

While regular exercise is generally associated with improved mental well-being, the result of this study demonstrates that the benefits of physical activity in rock-climbing may not shield individuals from mental health challenges. Several studies have examined climbing as a treatment of mood disorders, with promising results [23–25]. More elaborate trials are currently ongoing [26]. The effects of an intervention as a treatment of an established disorder, may not neccesarily, however, have equal primary preventing effects of the disorder itself. As mental health is influenced by a multitude of factors, including seasonal variations, further studies are needed to observe if results vary depending on these variables. As opposed to the general population, musculoskeletal injury has been shown to be an important determinant of mental health problems among athletes [8]. Injury among rock-climbers has been extensively researched in regard to both injury types and severity [27], but few studies have examined disability associated to musculoskeletal pain or dysfunction [28, 29]. Most studies have focused on rock-climbing specific injuries, and rock-climbing specific injury events [30]. The results of this study display a high self reported one-week prevalence of musculoskeletal problems, in locations known to be prone to injury in rock-climbers, but also considerable rates of problems in body-locations less examined, such as the back, knee and foot. The upper extremity had the highest rate of participants experiencing substantial problems (finger and hand – 16.5%, shoulder 11.0% and arm – 7.7%). The importance of recognizing the distinctive stressors, psychological demands, and individual experiences that rock-climbers face is evident. Since this study, due to its cross-sectional design, makes no effort to determine the association between stressors and mental ill health, further research is needed to determine if the risk factors and underlying cause for developing these symptoms differ between the rock-climbing and the general population. Musculoskeletal injury has been closely associated to mood disorders, and the possible relation between these among rock-climbing athletes need further mapping [8, 22].

This is one of the first studies examining the prevalence of general mental ill-health of rock-climbing athletes, and thus provides new insights into the health state of the athletes of this rapidly evolving sport. While the results displayed no differences in symptom prevalence between the rock-climbing and non-rock-climbing group, the high prevalence of depression, anxiety and stress of the rock-climbing group is concerning. The results display the need of a comprehensive perspective regarding injury, pain, dysfunction, and mental ill health among these athletes. The high prevalence of problems related to injury of both the advanced and elite athletes provides important insight for trainers and medical professionals working with athletes, bringing attention to the need of a broader perspective regarding the climbers’ general health. Mental health problems and injury among athletes are both treatable and preventable conditions, and early diagnosis significantly improves prognosis. Our results emphasize the urgent need to further map the extent and causes of mental health problems and co-occurent health problems, and to the sport community to take action and provide structured care and adequate support in order to come to terms with these issues.

### Limitations

While the study has several strengths in its novelty among the rock-climbing population, study design and sample size, there are several limitations. The methodology of the study with self-selection sampling is inherently a risk of sample bias, where under- or over-presentation from certain groups of participants is possible, both in the climbing and the control group. Also, no comparative injury data was collected from the control group, since the control group was not homogenously participating in a sport, and thus not suitable for collecting injury data. This challenges the interpretation of the injury prevalence. In addition, no attempts of risk factor analysis of the reported symptoms were made, due to the methodological limitations of the cross-sectional design of the study. To determine the potential risk factors for the high rates of mental ill health among rock-climbing athletes, studies of longitudinal design are needed.

## Conclusion

The overall results indicate high levels of symptoms of mental health problems and poor sleep quality in both rock-climbers and controls. Although no significant differences between the climbing group and the control group could be displayed, symptoms that warrant clinical attention is high. Overuse injuries were commonly reported among the rock-climbers in all examined injury locations. Previous studies reporting mental health problems to be more prevalent among athletes were contradicted in this study. The results display the need of a broader perspective regarding the climbers general health and the need to provide structured care and adequate support in order to come to terms with these concerns.

## Data Availability

The data that support the findings of this study are available from the corresponding author upon reasonable request.

## Abbreviations

DASS-21: Depression anxiety stress scale
OSTRC-O: The Oslo Sports Trauma Research Center Overuse Injury Questionnaire
PSQI: Pittsburg sleep quality index
IRCRA: International rock-climbing research association
BMI: Body mass index

## Footnotes

1 A “non-flash” ascent of a route refers to a successful ascent of a route where the climber has prior knowledge of the route or have carried out previous attempts to complete the route.
